# Sex Differences and Age Distributions in Invasive Treatments for Chalazion and Hordeolum in Japan: A 9-Year Nationwide Claims Study

**DOI:** 10.1016/j.xops.2026.101067

**Published:** 2026-01-09

**Authors:** Masamitsu Kido, Sayaka Kamada, Tomo Suzuki, Koji Kitazawa, Yasufumi Tomioka, Kengo Yoshii, Katsutoshi Shoda, Chie Sotozono

**Affiliations:** 1 Department of Orthopedic Surgery, Inage Hospital, Chiba, Japan; 2 Department of Ophthalmology, Kyoto Prefectural University of Medicine, Kyoto, Japan; 3 Department of Ophthalmology, Kyoto City Hospital, Kyoto, Japan; 4 Department of Mathematics and Statistics in Medical Sciences, Kyoto Prefectural University of Medicine, Kyoto, Japan; 5 Faculty of Medicine, First Department of Surgery, University of Yamanashi, Yamanashi, Japan

**Keywords:** Chalazion, Hordeolum, Invasive treatment, Database, Epidemiology

## Abstract

**Purpose:**

To analyze nationwide trends and demographic patterns of invasive treatments for chalazion (ITC) and invasive treatments for hordeolum (ITH) in Japan.

**Design:**

A retrospective population-based cohort study.

**Participants:**

Patients undergoing ITC or ITH procedures from 2014 to 2022.

**Methods:**

Using the National Database of Health Insurance Claims and Specific Health Checkups Open Data Japan, we calculated annual numbers and rates of ITC and ITH per 100 000 person-years. Sex-stratified and age-stratified demographic peak patterns were analyzed, and annual trends were evaluated using linear and Poisson regression models.

**Main Outcome Measures:**

Procedure rates, demographic distributions, and annual trends of ITC and ITH.

**Results:**

A total of 1 104 078 procedures (ITC: 465 379; ITH: 638 699) were recorded over the 9-year study period. The annual average rates were 40.9 for ITC and 56.2 for ITH per 100 000 person-years. Sex differences were prominent in younger groups: the overall female-to-male ratio was 1.1:1 for ITC and 1.0:1 for ITH, with a greater female predominance in youth (ITC: 2.0 at age 15–19 years; ITH: 1.7 at age 20–24 years). Invasive treatments for chalazion showed bimodal peaks in males (age 15–19 years: 40.9 procedures per 100 000 person-years; age 35–39 years: 56.0) and females (age 15–19 years: 80.6; age 30-34 years: 68.0). Invasive treatments for hordeolum showed bimodal peaks in males (age 10–14 years: 82.9; age 35-39 years: 72.1) and females (age 10–14 years: 102.4; age 30–34 years: 78.3). Both age-adjusted rates declined annually across all age groups and both sexes (*P* < 0.0167), except for an increase in 0–4-year-old ITC cases (*P* < 0.00088).

**Conclusions:**

This study reveals sex-related and age-related demographic patterns and an overall decline in invasive eyelid procedures over the past decade and provides valuable insights into the epidemiology of common eyelid diseases and surgical trends in Japan.

**Financial Disclosure(s):**

The authors have no proprietary or commercial interest in any materials discussed in this article.

Chalazion, a chronic meibomian gland granuloma, and hordeolum, an acute bacterial infection of eyelid glands, are among the most common ophthalmic conditions.^1^ Their clinical presentations often vary across demographic groups. For instance, pediatric chalazion can be recurrent and is associated with pediatric blepharokeratoconjunctivitis and amblyopia,^2^, ^3^, ^4^ while in elderly patients, distinguishing chalazion from eyelid malignancies is critical.^5^ Despite their clinical relevance, nationwide epidemiological data on chalazion and hordeolum remain scarce. To date, only 2 large-scale studies have reported on chalazion prevalence: one from India found rates of 0.95% in children and 0.51% in adults among >2 million new patients,^6^ while another study from the United States noted a 6.05% prevalence among older veterans.^7^ Notably, no population-based epidemiological data on hordeolum have been published to date.

To address these knowledge gaps, we utilized the National Database of Health Insurance Claims and Specific Health Checkups of Japan (NDB),^8^ a large-scale administrative dataset that captures >95% of the country's medical insurance claims. We previously demonstrated the utility of the NDB for epidemiological investigations of diagnostically challenging diseases by analyzing data derived from surgical procedures.^9^^,^^10^ This procedure-based approach, focusing on invasive treatments for chalazion (ITC) and invasive treatments for hordeolum (ITH), has been shown to improve the accuracy of database research by analyzing procedure codes rather than disease names.^11^ Although this method captures only more severe cases requiring surgical intervention, confirmation via macroscopic intraoperative findings strengthens diagnostic validity.

In this study, we aimed to elucidate the demographic characteristics (age and sex distributions) of patients undergoing ITC and ITH and to evaluate annual trends in the number of procedures performed across Japan. Furthermore, we sought to enhance understanding of the epidemiology underlying these common eyelid disorders.

## Methods

### Study Design and Data Sampling Technique

The study was conducted in accordance with the principles of the Declaration of Helsinki. According to the Japanese ethical guidelines, this study did not require Institutional Review Board approval or informed consent, as all data were obtained from publicly available sources. Moreover, to ensure patient anonymity, the publicly available “NDB Open Data Japan”^12^ database used in this study does not report data for procedures that occur <10 times annually.

Annual sex-stratified and age-stratified numbers (in 5-year increments) of ITC and ITH from 2014 through 2022 were extracted from the NDB Open Data Japan,^12^ an open-access subset of the NDB. The dataset included both outpatient and inpatient procedures. Demographic data from the Ministry of Internal Affairs and Communications^13^ were employed to compute the procedure rates per 100 000 person-years.

Procedure codes are used to identify ITC and ITH.

The following Japanese procedure codes were used to identify ITC and ITH:

(1) ITC (K214, K215): Removal of chalazion or partial eyelid resection for large chalazion. Invasive treatments for chalazion include the following 2 procedures:
  (a) K214: Removal of chalazion
  (b) K215: Partial eyelid resection (excision of large chalazion).
(2) ITH (K208): Incision of hordeolum (external or internal).

### Data Analyses

Over the 9-year study period, overall and age-stratified female-to-male (F/M) ratios were calculated. Demographic peak analyses of age-stratified procedure rates per 100 000 person-years were performed for males, females, and both sexes in order to illustrate the overall demographic burden across the Japanese population. To capture recent patterns, a detailed breakdown of the proportion of the 2 common eyelid procedures was obtained via the use of the most recent dataset from 2022. Additionally, the actual number of each procedure across the 10-year age groups was analyzed for the same year.

Annual trends were evaluated for both the number and rate of each procedure from 2014 to 2022. Procedure counts were analyzed using linear regression models. For procedure rates, Poisson regression models were fit with fiscal year entered as a continuous explanatory variable. Relative risks (RRs) were estimated for females, males, and the combined population, both overall and within age-stratified subgroups. In this context, an RR <1.0 indicated a year-on-year decrease in the procedure rate per 100 000 person-years, whereas an RR >1.0 indicated an increase.

### Statistical Analyses

The data were evaluated after adjusting for age by the direct method using the standard age structure derived from the 2015 Population Census in Japan, as previously reported.^14^ Annual trends in the number of procedures were evaluated using linear regression analysis.

Poisson regression models were constructed to examine the annual trends in the age-adjusted procedure rate per 100 000 population. The number of each procedure was set as the objective variable, and the observation time point (year) and sex were set as the explanatory variables. In addition, the population at each time point was considered by adding the person-year population to the model as an offset. The number was also adjusted for age by the direct method, using the same age structure.^14^ Moreover, annual trend analysis of the subgroups was performed using age-stratified and sex-stratified samples.

All statistical analyses were performed using the R version 3.6.2 Statistical Computing Program (R Foundation; www.r-project.org). Statistical significance was set at 2-sided *P* values of <0.05 for the linear regression models and <0.0167 (0.05/3) and <0.00088 (0.05/57) for the Poisson regression models for all age and age-stratified analyses, respectively, using the Bonferroni correction method for annual trend analysis.

## Results

### Comprehensive Analysis

The cumulative crude number of 2 procedures across all age groups and sexes amounted to 1 104 078 procedures (ITC: 465 379; ITH: 638 699) performed from 2014 through 2022. The annual average number rates for ITC and ITH, respectively, were 40.9 and 56.2 per 100 000 person-years. The overall F/M ratios were 1.1:1 for ITC and 1.0:1 for ITH.

### Mode Share and Proportion of Each Procedure in 2022

**Figure 1:**
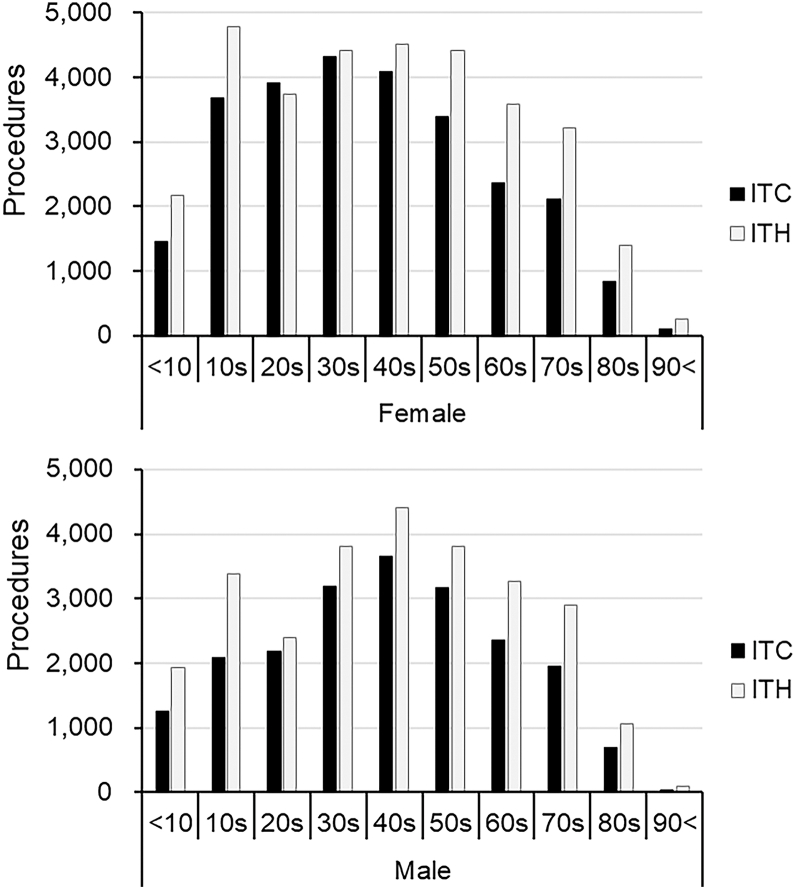
Number of procedures for ITC and ITH across 10-year age groups among females (upper) and males (lower) in 2022. A total of 106 704 procedures were performed (ITC: 47 083; ITH: 59 621). ITC = invasive treatments for chalazion; ITH = invasive treatments for hordeolum.

In 2022, the latest mode share of 2 common eyelid procedures was as follows: ITC (44.1%, 47 083/106 704) and ITH (55.9%, 59 621/106 704). To provide a detailed demographic breakdown of procedure utilization patterns, the actual number of procedures performed for each type across the 10-year age groups for females and males in 2022 is outlined in Figure 1.

### Demographic Peak Analysis during 2014 to 2022

**Figure 2:**
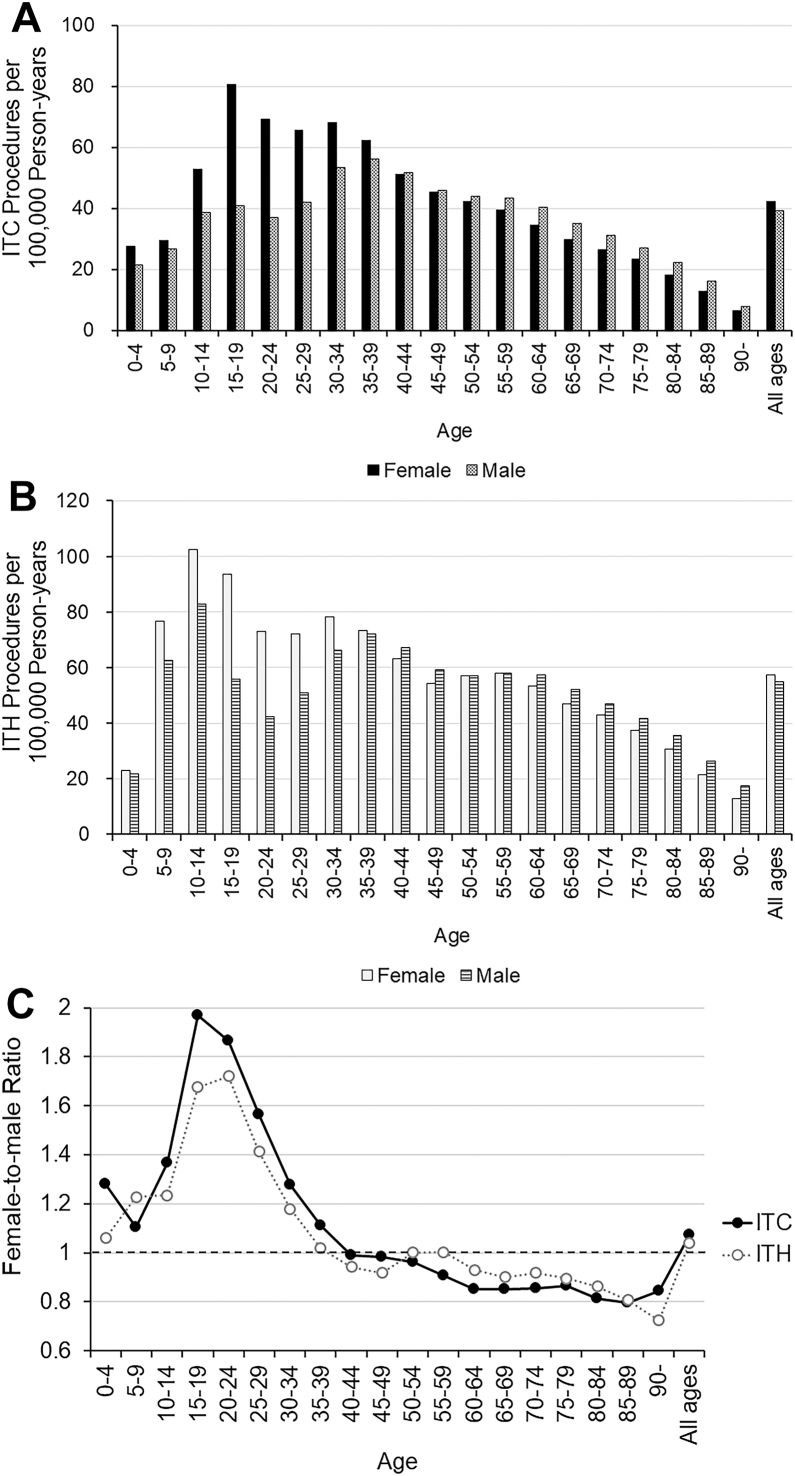
**A,** Age-stratified number rate of ITC procedures per 100 000 person-years in Japan (females and males, 2014–2022). **B,** Age-stratified number rate of ITH procedures per 100 000 person-years in Japan (females and males, 2014–2022). **C,** Age-stratified female-to-male ratio of ITC and ITH procedures (2014–2022). ITC = invasive treatments for chalazion; ITH = invasive treatments for hordeolum.

Throughout the study period, the age-stratified number rate of ITC exhibited bimodal peak patterns in females and males (Fig 2A). In females, peaks were identified at ages 15 to 19 years (major peak, 80.6 procedures per 100 000 person-years) and 30 to 34 years (minor peak, 68.0 procedures). In males, peaks were seen at ages 15 to 19 years (minor peak, 40.9 procedures) and 35 to 39 years (major peak, 56.0 procedures). Eventually, in both sexes, peaks were observed at ages 15 to 19 years (major peak, 121.5 procedures) and 30 to 34 years (major peak, 121.3 procedures). In the under 39-year-old age groups, female dominancy in the number rate was observed compared to male, while over 40, slight male dominancy was observed.

The age-stratified number of ITH also exhibited bimodal peak patterns in males and females (Fig 2B). In females, peaks were noted at ages 10 to 14 years (major peak, 102.4 procedures per 100 000 person-years) and 30 to 34 years (minor peak, 78.3 procedures). In males, peaks were observed at ages 10 to 14 years (major peak, 82.9 procedures) and 35 to 39 years (minor peak, 72.1 procedures). Eventually, in both sexes, peaks were observed at ages 10 to 14 years (major peak, 185.3 procedures) and 35 to 39 years (minor peak, 145.5 procedures). Similar to ITC, in the under 39-year-old age groups, female dominancy in the number rate was observed compared to male, while over 40, slight male dominancy was observed, except in subjects in their 50s.

The age-stratified F/M ratio for each procedure is shown in Figure 2C. A distinct peak in the F/M ratio was observed in the younger age group across the 2 procedures, with the highest ratios for ITC at 15 to 19 years (F/M ratio = 2.0) and for ITH at 20 to 24 years (F/M ratio = 1.7). In individuals aged ≥60 years, the F/M ratio decreased slightly, stabilizing at 0.7 to 0.9.

### Trends in the Annual Number from 2014 to 2022

**Figure 3:**
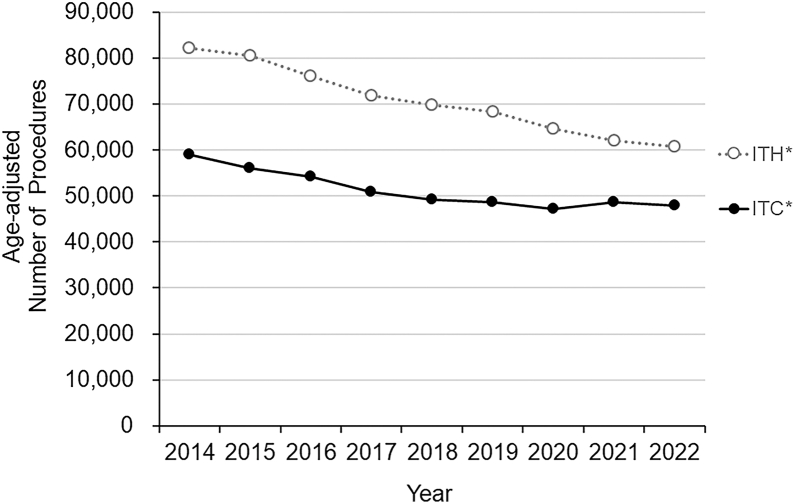
Trends in the annual age-adjusted number of ITC and ITH procedures in Japan from 2014 to 2022. ITC = invasive treatments for chalazion; ITH = invasive treatments for hordeolum. ∗Indicates a statistically significant trend (*P* < 0.05).

Over the 9-year study period, significant decreasing trends were observed in the age-adjusted number of ITC (*P* = 0.0005) and ITH (*P* < 0.0001) (Fig 3).

### Trends in the Annual Number Rate per 100 000 Person-Years from 2014 to 2022

**Figure 4:**
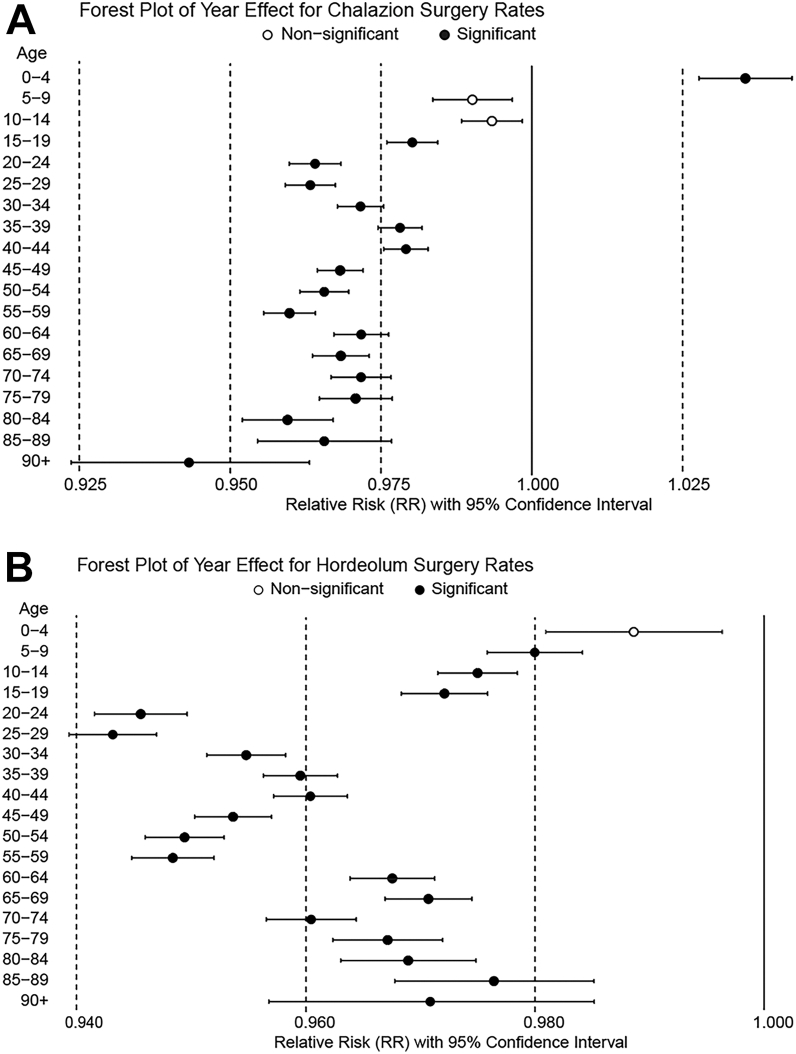
**A,** Relative risk of the age-stratified number of ITC procedures per 100 000 person-years for both sexes, using fitting Poisson regression models. **B,** Relative risk of the age-stratified number of ITH procedures per 100 000 person-years for both sexes, using fitting Poisson regression models. ITC = invasive treatments for chalazion; ITH = invasive treatments for hordeolum. Black dots represent significant and white dots represent nonsignificant.

The age-adjusted ITC number across all age groups per 100 000 person-years showed a decreasing annual trend for females, males, and both sexes (RR = 0.987, 0.959, and 0.973 for males, females, and both sexes, respectively; *P* < 0.0001) (Table 1). Subgroup analyses indicated decreasing trends in males aged 10-84 years and females aged 15-29 years and >45 years. Conversely, increasing trends were observed in males and females aged 0-4 years (*P* < 0.00088) (Fig 4A and Table S1, available at www.ophthalmologyscience.org).

**Table 1:** Poisson Regression Analysis of Annual Changes in Age-Adjusted ITC and ITH Procedure Rates per 100 000 Person-Years

Sex	Procedure	RR	95% CI (Low)	95% CI (High)	*P* Value
Female	ITC	0.987	0.985	0.988	<0.0001
	ITH	0.970	0.969	0.971	<0.0001
Male	ITC	0.959	0.957	0.960	<0.0001
	ITH	0.951	0.950	0.953	<0.0001
Female and male	ITC	0.973	0.972	0.975	<0.0001
	ITH	0.961	0.960	0.962	<0.0001

CI = confidence interval; ITC = invasive treatments for chalazion; ITH = invasive treatments for hordeolum; RR = relative risk.

Relative risk represents the rate ratio per 1-yr increase (time variable entered as continuous).

### Trends in the Annual Number Rate per 100 000 Person-Years from 2014 to 2022

The age-adjusted ITH number across all age groups per 100 000 person-years showed a decreasing annual trend for females, males, and both sexes (RR = 0.970, 0.951, and 0.961 for males, females, and both sexes, respectively; *P* < 0.0001) (Table 1).

Subgroup analyses showed decreasing trends in females and males across nearly all age groups (over 5 years), with stronger declines observed in males aged 20-59 years and females in their 20s (*P* < 0.00088) (Fig 4B and Table S2, available at www.ophthalmologyscience.org).

## Discussion

This observational epidemiological study analyzed the number of ITC and ITH procedures by sex and age using a comprehensive national medical claims database in Japan. To our knowledge, this is the first nationwide, population-based study to focus on surgical treatments for these eyelid diseases. By analyzing observational evidence data over a recent 9-year study period, we identified distinct sex-related and age-related patterns and an overall decline in ITC and ITH procedures.

Demographic analyses showed similar bimodal peak patterns for both ITC and ITH, with peaks in adolescence and early-to-mid adulthood, although with subtle differences (Fig 2A, B). Invasive treatments for hordeolum exhibited a major peak in early adolescence (early teens) and a minor peak in the 30s for both sexes. While the clinical prevalence of hordeolum in these age groups is well recognized, detailed epidemiological studies on its age distribution are scarce. The observed bimodal distribution of ITH may possibly be explained by multiple biological and behavioral factors. In adults, particularly men, elevated androgen levels and increased sebaceous and meibomian gland activity are known to enhance meibum secretion. In addition, systemic lipid metabolism abnormalities have been associated with meibomian gland dysfunction, even among younger and middle-aged adults.^15^^,^^16^ These physiological and lifestyle factors, including stress, sleep deprivation, and the use of cosmetics, may possibly disturb the eyelid margin environment and increase susceptibility to acute infection, collectively contributing to the bimodal age distribution observed in ITH. In contrast, ITC patterns differed by sex: females showed a major peak in the late adolescence and a minor peak in their late 30s, whereas males exhibited the opposite pattern. Although chalazion has long been considered a noninfectious condition, recent studies proposed *Cutibacterium acnes* (*C. acnes*) as a possible pathogen contributing to granuloma formation.^17^^,^^18^ Age-related changes in commensal bacteria, particularly the dominance of *C. acnes* in the meibomian glands of younger individuals,^19^ may influence the higher ITC prevalence in specific age and sex groups. Furthermore, meibomian gland orifice obstruction and meibum stagnation—known triggers for chalazion—tend to occur more frequently in premenopausal women.^20^^,^^21^ This may also contribute to the higher ITC prevalence in young females, although further investigation is needed.

The age-stratified female-to-male ratios showed peaks in the late teens to early 20s for both ITC and ITH (Fig 2C). This female predominance in younger age groups is consistent with findings from a previous case series among 206 chalazion cases^17^ and a retrospective observational case-control study of 18 238 Israeli residents,^22^ thus suggesting a potential role of sex hormones in disease pathogenesis. Interestingly, this sex difference pattern observed in ITC closely resembles that seen in meibomitis-related keratoconjunctivitis, a condition that frequently coexists with meibomian gland dysfunction and chalazion. Meibomitis-related keratoconjunctivitis^23^ and acne rosacea,^24^ which are both more common in young women, share clinical features and are associated with *C. acnes*, thus suggesting a potential common pathophysiological pathway. In addition to those biological mechanisms, hormonal changes during puberty may possibly affect meibomian gland function, predisposing young women to chalazion. Social and behavioral factors may also play a role. Frequent cosmetic and contact lens use, together with heightened cosmetic awareness among young women, may possibly increase the risk of eyelid gland obstruction and encourage earlier surgical intervention in this group.

The overall number and rates of ITC and ITH procedures showed significant declining trends over the 9-year study period (Fig 3 and Table 1). This general decrease might reflect improved facial hygiene^25^ and evolving beauty standards in Japan.^26^ In addition, although conservative treatments^27^ such as topical steroids,^28^ antibiotics, and hot compresses have long been available, their wider adoption during the study period may have further reduced the need for surgical intervention. Ophthalmologists may also have increasingly favored nonsurgical approaches as first-line management, reflecting evolving practice patterns. While intense pulsed light therapy^29^ has recently emerged as a novel option, it became available only toward the end of the study period and is unlikely to help explain the overall decline. Taken together, these clinical and societal factors likely contributed to the overall decline in invasive procedures. Additionally, detailed trend analyses of ITH rates per 100 000 person-years demonstrated decreasing rates across nearly all age groups for both sexes, with an even stronger decline among individuals in their 20s and 50s (Fig 4B). Interestingly, ITC showed a general decrease across most age groups, but an opposite increasing trend among children aged 0 to 4 years (Fig 4A). The increasing trend in that age group (ITC: *P* < 0.00088) is likely multifactorial. Expanded pediatric health care subsidies^30^^,^^31^ and improved screening may have encouraged earlier ophthalmic consultation and proactive treatment, while conservative management is often less feasible in very young children. Concerns about vision-related risks such as induced astigmatism and amblyopia may also have encouraged a more proactive approach among ophthalmologists. It also remains possible that the actual number of chalazion cases in that age group has increased. However, because our dataset does not allow further subdivision within the 0 to 4-year category, detailed interpretation of age-specific factors is limited, and further studies are needed to confirm these observations.

It should be noted that this study did have several limitations. First, our analysis focused on surgical procedures rather than the incidence of the diseases themselves. Discrepancies between surgical rates and disease incidence can arise from multiple factors, such as patient access to health care, evolving therapeutic paradigms, and individual physician preferences. The overall decline likely reflects higher surgical thresholds, while the rise in the 0 to 4 age group may indicate a tendency toward earlier intervention. Increased use of nonsurgical therapies also likely reduced the need for surgery. Second, procedural coding may overlap between ITC and ITH, and billing practices could vary among physicians and institutions. Finally, the generalizability of the findings to other countries is limited due to differences in health care systems, social structures, and population characteristics.

In conclusion, the findings in this nationwide demographic study revealed distinct patterns of sex differences and age distributions in invasive treatments for chalazion and hordeolum in Japan. Bimodal age peaks were observed in both sexes, and female predominance was more pronounced in younger age groups, particularly for ITC. In addition, the findings confirmed an overall decline in invasive eyelid procedures performed over the recent, nearly decade-long, study period. These findings provide valuable insights into the epidemiology of ITH and ITC in Japan and may contribute to a broader understanding of the underlying diseases.

## Declaration of Generative AI and AI-Assisted Technologies in the Writing Processs

During the preparation of this manuscript, the author (M.K.) used ChatGPT-4o mini to assist with translating the original Japanese draft into English. Subsequently, the authors reviewed and revised the content as needed and took full responsibility for the content of the published article.
