# Supplementary material for: Sex Differences and Age Distributions in Invasive Treatments for Chalazion and Hordeolum in Japan: A 9-Year Nationwide Claims Study
Source: Ophthalmol Sci. 2026 Jan 9;6(3):101067. doi: 10.1016/j.xops.2026.101067 (PMC12887806; doi:10.1016/j.xops.2026.101067)
Supplement: Tables S1 and S2 [file mmc1.pdf]

Supplementary Table 1 Poisson regression analysis of annual changes in age-stratified ITC procedure rates per 100,000 person-years.

**Sex**	**Age Group**	**RR**	**95% CI (low)**	**95% CI (high)**	***P*-value**
Female	0–4 years	1.038	1.028	1.048	<0.0001
	5–9 years	0.990	0.981	0.999	0.035
	10–14 years	1.008	1.001	1.015	0.019
	15–19 years	0.990	0.985	0.996	<0.0001
	20–24 years	0.973	0.967	0.978	<0.0001
	25–29 years	0.976	0.971	0.982	<0.0001
	30–34 years	0.993	0.988	0.998	0.009
	35–39 years	0.998	0.992	1.003	0.361
	40–44 years	0.997	0.991	1.002	0.229
	45–49 years	0.984	0.979	0.990	<0.0001
	50–54 years	0.980	0.975	0.986	<0.0001
	55–59 years	0.974	0.968	0.981	<0.0001
	60–64 years	0.986	0.979	0.993	<0.0001
	65–69 years	0.981	0.974	0.988	<0.0001
	70–74 years	0.979	0.972	0.986	<0.0001
	75–79 years	0.975	0.967	0.983	<0.0001
	80–84 years	0.958	0.948	0.969	<0.0001
	85–89 years	0.961	0.946	0.975	<0.0001
	≥90 years	0.944	0.921	0.967	<0.0001
Male	0–4 years	1.032	1.021	1.044	<0.0001

	5–9 years	0.990	0.981	1.000	0.045
	10–14 years	0.975	0.967	0.982	<0.0001
	15–19 years	0.961	0.954	0.968	<0.0001
	20–24 years	0.949	0.942	0.956	<0.0001
	25–29 years	0.945	0.938	0.951	<0.0001
	30–34 years	0.946	0.940	0.952	<0.0001
	35–39 years	0.958	0.952	0.963	<0.0001
	40–44 years	0.962	0.957	0.967	<0.0001
	45–49 years	0.953	0.948	0.958	<0.0001
	50–54 years	0.952	0.946	0.957	<0.0001
	55–59 years	0.947	0.941	0.953	<0.0001
	60–64 years	0.959	0.953	0.965	<0.0001
	65–69 years	0.957	0.951	0.963	<0.0001
	70–74 years	0.965	0.958	0.972	<0.0001
	75–79 years	0.966	0.957	0.974	<0.0001
	80–84 years	0.960	0.949	0.971	<0.0001
	85–89 years	0.971	0.954	0.989	0.002
	≥90 years	0.940	0.903	0.978	0.002
Female&Male	0–4 years	1.035	1.028	1.043	<0.0001
	5–9 years	0.990	0.984	0.997	0.004
	10–14 years	0.993	0.988	0.998	0.010
	15–19 years	0.980	0.976	0.984	<0.0001
	20–24 years	0.964	0.960	0.968	<0.0001
	25–29 years	0.963	0.959	0.967	<0.0001

	30–34 years	0.972	0.968	0.975	<0.0001
	35–39 years	0.978	0.975	0.982	<0.0001
	40–44 years	0.979	0.975	0.983	<0.0001
	45–49 years	0.968	0.964	0.972	<0.0001
	50–54 years	0.966	0.962	0.970	<0.0001
	55–59 years	0.960	0.956	0.964	<0.0001
	60–64 years	0.972	0.967	0.976	<0.0001
	65–69 years	0.968	0.964	0.973	<0.0001
	70–74 years	0.972	0.967	0.977	<0.0001
	75–79 years	0.971	0.965	0.977	<0.0001
	80–84 years	0.960	0.952	0.967	<0.0001
	85–89 years	0.966	0.955	0.977	<0.0001
	≥90 years	0.943	0.924	0.963	<0.0001

RR, relative risk; CI, confidence interval; ITC, invasive treatments for chalazion.

RR represents the rate ratio per one-year increase (time variable entered as continuous).

Supplementary Table 2 Poisson regression analysis of annual changes in age-stratified ITH procedure rates per 100,000 person-years.

**Sex**	**Age Group**	**RR**	**95% CI (low)**	**95% CI (high)**	***P*-value**
Female	0–4 years	0.988	0.977	0.999	0.032
	5–9 years	0.984	0.978	0.990	<0.0001
	10–14 years	0.977	0.973	0.982	<0.0001
	15–19 years	0.981	0.976	0.986	<0.0001
	20–24 years	0.948	0.943	0.953	<0.0001
	25–29 years	0.952	0.947	0.958	<0.0001
	30–34 years	0.964	0.960	0.969	<0.0001
	35–39 years	0.972	0.967	0.977	<0.0001
	40–44 years	0.972	0.968	0.977	<0.0001
	45–49 years	0.964	0.959	0.969	<0.0001
	50–54 years	0.963	0.958	0.967	<0.0001
	55–59 years	0.963	0.958	0.969	<0.0001
	60–64 years	0.982	0.977	0.987	<0.0001
	65–69 years	0.982	0.976	0.987	<0.0001
	70–74 years	0.968	0.962	0.973	<0.0001
	75–79 years	0.973	0.967	0.980	<0.0001
	80–84 years	0.976	0.968	0.984	<0.0001
	85–89 years	0.976	0.964	0.987	<0.0001
	≥90 years	0.975	0.958	0.992	0.004
Male	0–4 years	0.989	0.978	1.000	0.054

	5–9 years	0.975	0.969	0.981	<0.0001
	10–14 years	0.972	0.967	0.977	<0.0001
	15–19 years	0.958	0.952	0.964	<0.0001
	20–24 years	0.941	0.935	0.948	<0.0001
	25–29 years	0.931	0.925	0.937	<0.0001
	30–34 years	0.944	0.939	0.949	<0.0001
	35–39 years	0.947	0.943	0.952	<0.0001
	40–44 years	0.950	0.945	0.954	<0.0001
	45–49 years	0.944	0.940	0.949	<0.0001
	50–54 years	0.937	0.932	0.941	<0.0001
	55–59 years	0.933	0.928	0.938	<0.0001
	60–64 years	0.954	0.949	0.959	<0.0001
	65–69 years	0.960	0.955	0.965	<0.0001
	70–74 years	0.953	0.947	0.959	<0.0001
	75–79 years	0.960	0.953	0.967	<0.0001
	80–84 years	0.959	0.951	0.968	<0.0001
	85–89 years	0.976	0.962	0.990	0.001
	≥90 years	0.958	0.933	0.983	0.001
Female&Male	0–4 years	0.989	0.981	0.996	0.004
	5–9 years	0.980	0.976	0.984	<0.0001
	10–14 years	0.975	0.972	0.978	<0.0001
	15–19 years	0.972	0.968	0.976	<0.0001
	20–24 years	0.946	0.942	0.950	<0.0001
	25–29 years	0.943	0.939	0.947	<0.0001

	30–34 years	0.955	0.951	0.958	<0.0001
	35–39 years	0.960	0.956	0.963	<0.0001
	40–44 years	0.960	0.957	0.964	<0.0001
	45–49 years	0.954	0.950	0.957	<0.0001
	50–54 years	0.949	0.946	0.953	<0.0001
	55–59 years	0.948	0.945	0.952	<0.0001
	60–64 years	0.968	0.964	0.971	<0.0001
	65–69 years	0.971	0.967	0.975	<0.0001
	70–74 years	0.960	0.957	0.964	<0.0001
	75–79 years	0.967	0.962	0.972	<0.0001
	80–84 years	0.969	0.963	0.975	<0.0001
	85–89 years	0.976	0.968	0.985	<0.0001
	≥90 years	0.971	0.957	0.985	<0.0001

RR, relative risk; CI, confidence interval; ITH, invasive treatments for hordeolum.

RR represents the rate ratio per one-year increase (time variable entered as continuous).
